# The Sources of Wastage from Disease in the A. E. F.

**Published:** 1918-09

**Authors:** 


### THE SOURCES OF WASTAGE FROM DISEASE IN THE A. E. F.

Brigadier-General Ireland, Chief Surgeon of the American Expeditionary Forces, was not scheduled as one of the speakers, but was asked to introduce Major Emerson whom he had detailed to discuss "The Sources of Wastage from Disease in the American Expeditionary Forces". General Ireland said the present method of reporting the sick and wounded of the American Expeditionary Forces was modeled after the system in effect by the Medical Departments of the British Armies in France, and that all the records, which are now almost 100 % for the A. E. F. for the year ending May 31st, 1918, have been tabulated with the aid of the recently installed Hollerith machines. The information for this first year's experience is now available by diseases, by months, giving the cases among officers, men and civilians, the deaths among these groups, the total days lost in hospital and in quarters, and the disposal of each case by death, return to duty, discharge or otherwise; and that from June 1918 forward it will be possible to give in addition the data as to the organization of the patient. The information is now tabulated daily and can be presented for any unit of time by grouping the daily reports. It will be particularly valuable now to compare our results with those of the corresponding seasons of a year ago when organization of preventive sanitation had made but little progress.

General Ireland further said that it is the expectation of the office of the " Sick and Wounded " not only to accumulate necessary records, and transmit them to the Surgeon General in Washington, but to make such immediate practical use of the daily reports, as soon as they are received, as will aid in the sanitary control and prevention of disease, and serve to assist and guide those chiefly occupied with the treatment of the sick. He concluded by saying that " a medical officer is judged by his morbidity and not by his mortality rates. To reduce non-effectiveness from disease to the lowest possible point is the immediate responsibility of the medical department. "

Major Haven Emerson in discussing " Wastage in the A. E. F. " gave a lot of interesting and suggestive data fresh from the new statistical machine in the Chief Surgeon's Office. He said that for a given period, prior to May 21, 62,714,000 days were spent by American soldiers in France. If the Germans desire any further proof of the fact that the United States has an army in France, besides the reception that the Prussian Guards and other picked troops received at Cantigny, Château-Thierry, and several other historic spots where the Huns have met the Americans, they should be provided with actual statistics. Statistics show that on account of sickness our troops lost or wasted 1,482,000 days of service. The problem, therefore, is what would the Americans have done to the Germans if they had not been so sickly? Now then, it is proposed to prevent these 1,481,000 days of sickness in the A. E. F., which might be expected for the next year; and with American troops coming over since June 1 at the rate of more that 300,000 per month, and with the new draft act that provides for 13,500,000 more United States soldiers, it is quite possible that in a year or two the Kaiser may suspect that the Americans are fighting with the French and British.

These figures, however, are not quite as formidable as facetious minds would make them appear, because, according to the new statistical machine, the soldiers lost only 2.377 o/o of total possible days' service. It is, therefore, assumed that they were fit for fighting for the other 97.623 o/o of their time — a good " batting " average. The fact is, as Major Emerson pointed out, the health of the American troops in France has been remarkably good, much better than that of the same men in the United States, but this does not mean that a stillqetter record may not be made this year.

Mumps was the one greatest source of wastage of the youths of the American army ; bronchitis caused the second greatest loss of time ; venereal diseases ranked third ; laryngitis, pharyngitis, and tonsilitis ranked fourth, lobar pneumonia fifth ; measles was sixth, but broncho-pneumonia, which was so prevalent among the soldiers in the cantonments in the United States, stood thirteenth on the list. The other diseases which caused wastage in the A. E. F. are given by Major Emerson in the order of their importance.

The value of morbidity and mortality statistics lies in pointing out the prevailing diseases in order that those which are preventable may be reduced, or eradicated. Major Emerson in his address not only gave the exact figures regarding diseases that have caused wastage in the A. E. F., but also showed what was necessary to be done to combat them. His paper should be read by every medical officer in the A. E. F., each of whom should strive every day to do his part in eradicating preventable diseases among the soldiers who are fighting to make the world a better place in which to live.

